# Aflatoxicosis in African greater cane rats (*Thryonomys swinderianus*)

**DOI:** 10.14202/vetworld.2018.1001-1005

**Published:** 2018-07-27

**Authors:** Henry O. Jegede, Ahmed O. Akeem, Oluwafemi B. Daodu, Afolabi A. Adegboye

**Affiliations:** 1 Zoo/Wildlife Unit, Veterinary Teaching Hospital, University of Ilorin, Ilorin, Nigeria; 2 Department of Veterinary Microbiology, University of Ilorin, Ilorin, Nigeria; 3 Department of Veterinary Pathology, University of Ilorin, Ilorin, Nigeria

**Keywords:** aflatoxicosis, African greater cane rat, management, pathology, *Thryonomys swinderianus*

## Abstract

**Aim:**

Aflatoxicosis is a widespread problem in captive animals fed on stored food and has been reported in various animals both domestic and wild. This report documents the clinicopathologic, microbial diagnostic findings and therapeutic regime for a study on the presentation, management, and outcome of aflatoxicosis in greater cane rats.

**Materials and Methods:**

A total of 65 greater cane rats suspected to be exposed to the toxin were examined clinically along with their environment. Feed samples, recently deceased carcasses and some moribund carcasses were collected for the study. Carcasses were subjected to gross and histopathologic investigations while feed and organs were subjected to microbiological investigations.

**Results:**

Gross lesions included hepatic lipidosis with ecchymotic hemorrhages, distended gallbladder, and renomegaly with ecchymosis among others. Histopathology revealed loss of hepatocellular architecture with massive centrilobular hepatocyte necrosis and diffuse steatotic damage characterized by macrovacuoles. Other histologic findings included pulmonary congestion, moderate renal tubular degeneration, and necrosis of epithelial tubular cells. *Aspergillus flavus* was isolated from the feed and ingesta. Total aflatoxin detected in feed sample was found to be over 400 ppm. *Klebsiella* species, *Staphylococcus* species, and *Bacillus* species were isolated from the liver and intestinal content. Management was attempted using Fungizal^®^ (Avico, Jordan) (which contains Thymol, benzoic acid, sorbic acid, and kaolin) and Orego-Stim^®^ (Saife, USA) (which contains carvacrol and thymol) which were instituted in feed and Superliv^®^ (Ayurvet, India) (polyherbal) liquid was instituted in water for 5 days at manufacturers’ dosage. All clinical signs disappeared, and no more deaths were recorded following management.

**Conclusion:**

This report concludes that aflatoxicosis causes severe mortality in greater cane rats and can be prevented and managed successfully.

## Introduction

The greater cane rat (*Thryonomys swinderianus*) is a wild animal recently domesticated in the past 40 years for meat production in several Sub-Saharan African countries [1]. It is a member of one of two species of cane rats, a small family of African hystricognath rodents [2].

With the rapidly increasing domestication and farming of this species, various diseases and husbandry problems are emerging mostly due to poor management practices while others due to the nature of the rodents [3,4].

Aflatoxins are a group of closely related and extremely toxic mycotoxins. They are produced primarily by *Aspergillus flavus* and *Aspergillus parasiticus* and can occur as natural contaminants of foods and feeds [5]. Toxigenic strains of *A. flavus* and *A parasiticus* on peanuts, soybeans, corn (maize), and other cereals produce aflatoxin either in the field or during storage when moisture content and temperatures are sufficiently high for fungal growth (consistent day and night temperatures>70°F) [6]. Aflatoxins have been shown to be hepatotoxic, carcinogenic, mutagenic, and teratogenic to different species of animals [7,8].

Following many reports of vast mortality in local cane rat farms with suspected aflatoxicosis outbreak, the authors decided to investigate a farm also suffering from these mass mortalities and report the findings in this paper.

## Materials and Methods

### Ethical approval

The experiment was carried out in accordance with the guidelines laid down by the University of Ilorin, Veterinary Teaching Hospital Animal Ethics Committee, file number VTH/AE/001/2016.

### Study animals

The farm studied is located in Kwara state, North Central region of Nigeria with an initial population of 65 rats before the study. Feed samples and recently deceased (<10 h) carcasses and some moribund carcasses were collected for the study. Clinical observations were performed, and environmental monitoring equipment was fixed in feed stores.

### Necropsy and histopathology

The two moribund rats were sacrificed and added with the 8 recently dead carcasses. A detailed postmortem examination was carried out on the carcasses. Gross pathological lesions were observed, documented and photographed. Samples of the liver, lung, and kidney showing abnormal gross lesions were collected and fixed in 10% buffered neutral formalin for a minimum of 48 h. The tissues were then trimmed, histosettes and dehydrated in graded concentrations of alcohol (70%, 80%, 90%, and 100%) using an automatic tissue processor (Kedee KD-TS6A, China), following fixation. The tissues were cleared with xylene, embedded in molten paraffin wax block, and labeled appropriately [9]. The tissue paraffin blocks were sectioned at 5 μm thick, using a rotary microtome (Kedee MR2258S, China). The sectioned tissues were mounted on a clean glass slide, dried at room temperature, stained with hematoxylin and eosin stains and a coverslip placed on the sections to prevent damage. The slides were assessed using a light microscope at 40× and 100× objective lenses to determine any histopathological changes. Photomicrographs of lesions from the liver, lung, and kidney were taken, transferred to a computer and labeled appropriately.

### Microbiology

Samples of liver, spleen, kidney, and lungs, intestinal content, and feed were aseptically collected, properly labeled, and kept in a cool box containing ice packs. The samples were transported, within 2 h, to Veterinary Microbiology Laboratory, University of Ilorin, for analyses.

About 10 g of each sample was pre-enriched in 90 ml of buffered peptone water and incubated at 37°C for 18-24 h [10]. Pre-enriched samples were subcultured on blood agar (Oxoid, UK) and Sabouraud dextrose agar (SDA) (Oxoid, UK) and incubated at 37°C and 25°C for 24 h and 3-4 days, respectively, for bacterial and fungal isolation. Bacterial growth on blood agar was subjected to biochemical reactions which include Gram staining, catalase, oxidase, Triple sugar iron, citrate utilization, and urease [11] while the fungal growth on SDA was identified using lactophenol cotton blue staining procedure [12]. Total bacterial count for the feed (1%) was carried out using the surface plating method.

### Aflatoxin quantification of feed

Feed samples were analyzed by competitive enzyme-linked immunosorbent assay (cELISA) using AgraQuant^®^ total aflatoxin assay 4/40 kit (Romer Lab Singapore Pte. Ltd.). Aflatoxin extraction, detection, and quantification were carried out by standard methods [13], as explained in an earlier report.

### Management

Management was attempted using Fungizal^®^ (Avico, Jordan) (antifungal and aflatoxin binder containing thymol, benzoic acid, sorbic acid, and kaolin) and Orego-Stim^®^ (Saife, USA) (carvacrol and thymol) were instituted in feed, and Superliv^®^ (Ayurvet, India) (polyherbal) liquid was instituted in water for 5 days at manufacturers’ dosage which is 2 kg per ton of feed, 1 kg per ton of feed, and 1 ml per liter of water, respectively.

## Results

Mean environmental parameters for the period was conditions of about 57.3% humidity and temperature of 29.9°C.

8 carcasses of recently deceased and 2 moribund greater cane rats were necropsied. 24 of 65 (40% mortality) cane rats from the group had previously died before the study. Clinical observations revealed inactivity, respiratory distress, and extension of forelimbs and hindlimbs in prone position before death. Diet consisted of a self-formulated feed predominated by maize and groundnut cake which was constituted and stored onsite.

### Postmortem findings

#### Gross pathology

**Figure-1:**
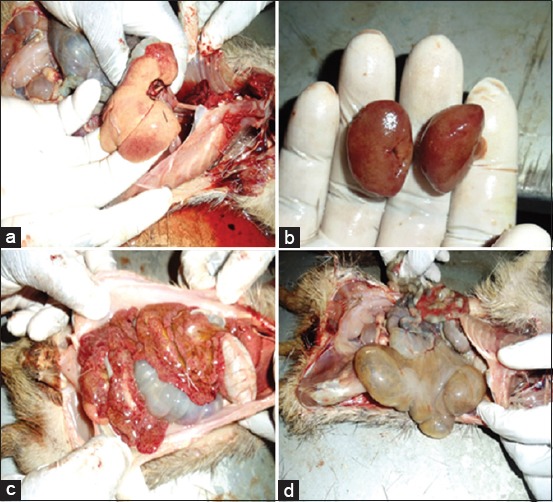
Gross lesions: (a) Hepatic lipidosis with areas of petechiation, (b) renomegaly with ecchymoses, (c) extensive inflammation of omental fat/peritonitis, and (d) ballooned cecum.

Necropsies were performed on 10 rats, and all animals were in good postmortem condition. Postmortem findings include hepatic lipidosis with ecchymotic hemorrhages (9/10) (Figure-1a), distended gallbladder (10/10). Kidney was also enlarged with ecchymosis (8/10) (Figure-1b). Bloodstain was found around the prepuce of one of the males (1/2) devoid of any visible injuries. Other lesions include pulmonary consolidation (7/10), pulmonary congestion (7/10), generalized peritonitis, omentitis (5/10) (Figure-1c), and lymphadenitis and cecal dilatation (7/10) (Figure-1d).

#### Histopathology

**Figure-2:**
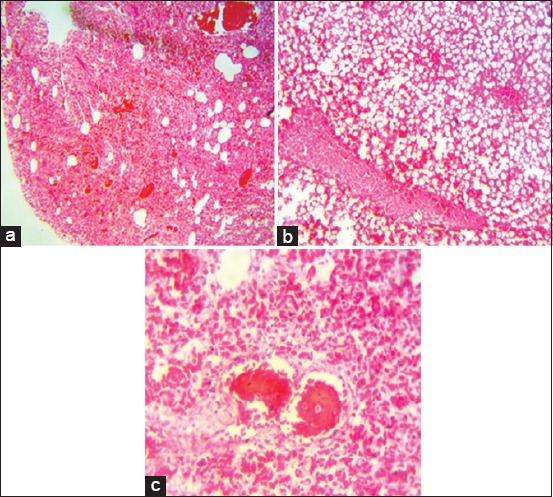
Photomicrographs showing panoramic views of (a) lung section 10× showing alveolar congestion, (b) liver section 40× showing loss of architecture and diffuse hepatic lipidosis, and (c) kidney section 40× showing tubular epithelium necrosis and eosinophilic infiltrations in renal parenchyma H and E.

Histopathological evaluation revealed loss of hepatocellular architecture showing massive centrilobular necrosis with diffuse steatotic damage characterized by macrovacuoles which were diffusely distributed (Figure-2a). Alveolar congestion was seen in the lungs with distention of blood vessels (Figure-2b). In the kidneys, moderate parenchymatous tubular degeneration and necrosis of tubular epithelial cells were present. There were also severe eosinophilic infiltrations in the renal parenchyma (Figure-2c).

### Microbiology results

*A. flavus* was isolated from the feed and ingesta. *Klebsiella* species, *Staphylococcus* species and *Bacillus species* were isolated from the liver and intestinal content. The feed had a total bacterial count of 2.5×10^16^ CFU/1 ml of 1% rat feed.

Total aflatoxin detected in feed samples was found to average over 400 ppm.

### Management outcome

All clinical signs had disappeared, and no more deaths were recorded following treatment, although about 10 more cane rats had died before treatment commenced.

## Discussion

Aflatoxicosis is a widespread problem in captive animals fed on stored food [14-16], but there has been no published report of this disease in the greater cane rat to the knowledge of the authors. The incriminating compounds mostly reported, to be contaminated with aflatoxins in Nigeria include maize [13,17] and groundnuts [18], which were the main ingredients of the poorly stored feed of the cane rats in this report.

There are no regulatory laws known on the allowed aflatoxin levels of feed in Nigeria, but the United States food and drug administration recommend a maximum permissible aflatoxin level of 20 ppm in corn, peanut products, other animal feeds, and feed ingredients for immature or dairy animals or when the intended use is not known [19], over 20 times this level was detected in the feed of these reported rats.

The gross lesions seen in these rats were most pronounced in the liver, and these findings were reported earlier in a murine model [20,21], in Mink [22] and New Zealand White rabbits [23]. The gross lesions observed in the gallbladder corroborated by previous author [23], who reported similar lesions in rabbits experimentally induced with aflatoxicosis. In addition, the gross lesions observed in the kidney of these rats are in agreement with a report in albino rats [21]. Similar hepatic and renal gross findings were also reported in poultry [24,25]. All gross pathologies were similar to those earlier reported except omentitis and cecal dilation which may be due to secondary bacterial infection from the other listed isolates, especially with the high bacterial load of the feed or also due to the prolonged prostration of the rats. Histologic lesions were also consistent with previous reports in rats, rabbits, mink, and poultry [21-23,26], although no literature reported findings of aflatoxicosis in greater cane rats.

The bacteria isolated from the ingesta in the present case, i.e., *Staphylococcus* species, *Bacillus* species, and *Klebsiella* species were not thought to have played significant roles in the gross and histopathology observed, as these organisms have been reported in previous work as being normal gastrointestinal flora of this rat, although may cause food poisoning in man especially the toxins produced by *Bacillus* (*Bacillus cereus*) [4,27,28].

When there is inadequate monitoring of environmental conditions of storage, Aspergillus levels in diet will foster in the warm and humid storage since synthesis of aflatoxins in feeds are increased at temperatures above 27°C humidity levels >62% and moisture levels in the feed above 14% [29].

Superliv liquid contains herbs such as *Andrographis paniculata, Azadirachta indica*, *Boerhaavia diffusa, Eclipta alba, Tinospora cordifolia*, and many others some of which have been shown to possess antioxidant, hepatoprotective, immunomodulatory, and or immunostimulatory activities in rodents [30,31]. Thymol and carvacrol, which are active ingredients in Fungizal and Orego-Stim feed additives, have been reported to inhibit the growth and aflatoxin production by *A. flavus* [32]. Thymol has also been reported to have ameliorative effects on hepatic oxidative stress injury [33]. Based on these findings, an educated decision was reached to select the cocktail as a treatment regime for this disease.

Extensive liver damage, as in these cases, can lead to reduced clotting factor synthesis with subsequent acute to chronic hemorrhage [6,34] and could be the reason for the blood stain found on the prepuce of males.

## Conclusion

This paper describes acute aflatoxicosis in greater cane rats causing severe mortality in a captive setting which could also include zoos and other private collections, and toxin binders and herbal hepatoprotective agents may be helpful for treatment. Feed should be stored properly by cooling and drying operations combined with ventilation, which are necessary to maintain proper and uniform temperature levels (<26°C) and to minimize humidity (<50%); and if possible avoid groundnut or cottonseed cake in feed formulation for these animals, alongside standard atmospheric condition monitoring for both rats and feed.

## Authors’ Contributions

HOJ and AOA conceptualized and designed the study, wrote the protocol, and wrote the first draft of the manuscript. OBD contributed in the literature search, data analyses, and interpretation of results. AAA assisted in the acquisition of data by laboratory techniques. All authors read and approved the final manuscript.
